# The effect of endothelin a receptor inhibition and biological sex on cutaneous microvascular function in non‐Hispanic Black and White young adults

**DOI:** 10.14814/phy2.16149

**Published:** 2024-07-17

**Authors:** Casey G. Turner, Matthew J. Hayat, Jeffrey S. Otis, Arshed A. Quyyumi, Brett J. Wong

**Affiliations:** ^1^ Department of Kinesiology and Health Georgia State University Atlanta Georgia USA; ^2^ Molecular Cardiology Research Institute Tufts Medical Center Boston Massachusetts USA; ^3^ School of Public Health Georgia State University Atlanta Georgia USA; ^4^ Emory Clinical Cardiovascular Research Institute Emory University School of Medicine Atlanta Georgia USA

**Keywords:** biological sex, endothelin‐1, endothelium, microcirculation, nitric oxide

## Abstract

The purpose of this study was to investigate whether endothelin‐A receptor (ET_A_R) inhibition in non‐Hispanic Black (NHB) and White (NHW) young adults depends on biological sex. We recruited females during low hormone (*n* = 22) and high hormone (*n* = 22) phases, and males (*n* = 22). Participants self‐identified as NHB (*n* = 33) or NHW (*n* = 33). Participants were instrumented with two microdialysis fibers: (1) lactated Ringer's (control) and (2) 500 nM BQ‐123 (ET_A_R antagonist). Local heating was used to elicit cutaneous vasodilation, and an infusion of 20 mM L‐NAME to quantify NO‐dependent vasodilation. At control sites, NO‐dependent vasodilation was lowest in NHB males (46 ± 13 %NO) and NHB females during low hormone phases (47 ± 12 %NO) compared to all NHW groups. Inhibition of ET_A_R increased NO‐dependent vasodilation in NHB males (66 ± 13 %NO), in both groups of females during low hormone phases (NHW, control: 64 ± 12 %NO, BQ‐123: 85 ± 11 %NO; NHB, BQ‐123: 68 ± 13 %NO), and in NHB females during high hormone phases (control: 61 ± 11 %NO, BQ‐123: 83 ± 9 %NO). There was no effect for ET_A_R inhibition in NHW males or females during high hormone phases. These data suggest the effect of ET_A_R inhibition on NO‐dependent vasodilation is influenced by biological sex and racial identity.

## 1 INTRODUCTION

The prevalence of total cardiovascular diseases (CVD) and hypertension, a risk factor for CVD, is greater in the non‐Hispanic Black (NHB) adults relative to the non‐Hispanic White (NHW) adults (Tsao et al., 2023). There is also a notable sex difference in the prevalence of CVD in the United States overall (males: 52%, females: 45%), but this sex difference is abrogated in NHB adults (males: 59%, females: 59%) (Tsao et al., 2023). Further understanding of the mechanisms underlying this disparity is necessary to improve cardiovascular health outcomes in the NHB males and females.

Cardiovascular health, in part, depends on a balance between dilator and constrictor mechanisms in the vasculature (Spieker et al., 2006). NHB individuals often display an imbalance in these mechanisms, characterized by blunted vasodilator (Campia et al., 2002; Cardillo et al., 1998; Hurr et al., 2018; Kim et al., 2018; Melikian et al., 2007; Miller et al., 2021; Morris et al., 2013; Ozkor et al., 2014; Patik et al., 2018; Pienaar et al., 2014; Strain et al., 2005; Turner et al., 2020; Wong et al., 2020) and enhanced vasoconstrictor (Adefurin et al., 2013; Ray & Monahan, 2002; Sherwood et al., 2017; Stein et al., 2000; Thomas et al., 2006; Vranish et al., 2018) responses (Brothers et al., 2019). Data from our lab, and others, have shown reduced microvascular vasodilation in young, healthy NHB adults (Turner et al., 2023; Hurr et al., 2018; Kim et al., 2018; Miller et al., 2021; Patik et al., 2018; Turner et al., 2020; Wolf et al., 2020; Wong et al., 2020). The endothelium produces several molecules that affect vascular health. Two important endothelial‐derived molecules are nitric oxide (NO), a cardioprotective vasodilator when produced via endothelial NO synthase (eNOS) (Vallance & Chan, 2001), and endothelin‐1 (ET‐1), a powerful vasoconstrictor (Pollock & Pollock, 2005). Increased ET‐1 signaling is associated with reduced eNOS protein expression and NO synthesis and increased oxidative stress (Loomis et al., 2005; Ramzy et al., 2006; Sud & Black, 2009; Wedgwood et al., 2001; Wedgwood & Black, 2005). Similarly, NHB adults display reduced NO bioavailability (Kalinowski et al., 2004; Melikian et al., 2007; Ozkor et al., 2014; Turner et al., 2020), reduced NO‐dependent vasodilation (Hurr et al., 2018; Kim et al., 2018; Miller et al., 2021; Patik et al., 2018; Wong et al., 2020), and increased oxidative stress (Deo et al., 2015; Feairheller et al., 2011; Hurr et al., 2018; Kalinowski et al., 2004; Morris et al., 2012). The vasoconstrictor actions of ET‐1 are largely mediated by the binding of ET‐1 to the endothelin‐A receptor (ET_A_R) subtype on vascular smooth muscle cells (VSMC) (Verhaar et al., 1998).

We recently demonstrated that ET_A_R inhibition can augment cutaneous NO‐dependent vasodilation in NHB young adults, and this effect is independent of superoxide; however, we tested both females and males and were not powered to assess potential effects of biological sex (Turner, Hayat, Grosch, Quyyumi, et al., 2023). Although recent data (Akins et al., 2022) suggests ET_A_R inhibition can improve microvascular endothelial function in young NHB females, there are still a few unresolved questions. First, females were only tested during the low hormone phase of the natural menstrual cycle or oral contraceptive pill (OCP) use (Akins et al., 2022), and it is unknown whether similar responses are observed in females during high hormone phases. This is an important unresolved question, as data suggests estradiol may influence the balance between ET_A_R and the endothelin‐B receptor subtype (ET_B_R), which can elicit vasodilation (Sebzda et al., 2018; Shoemaker et al., 2021), to favor activation of ET_B_R. This suggests that ET_A_R activity may be increased during low estrogen phases, and consequently reduce endothelium‐dependent and/or NO‐dependent vasodilation during this phase, but this has yet to be directly assessed, and the effect of racial identity on this relationship is unknown. Second, it is unknown how responses to ET_A_R inhibition in females during both low and high hormone phases compare to responses in males. Previous studies suggest that mechanisms underlying reduced NO‐dependent vasodilation may differ between young Black females and males (Patik et al., 2018). Further, recent data suggests microvascular function is reduced in NHB females relative to NHW females across three distinct phases of the natural menstrual cycle, positively correlating with circulating estradiol levels (D'Agata et al., 2021), but further mechanisms associated with these differences were not investigated.

The purpose of this cross‐sectional study was to determine whether the effect of ET_A_R antagonism on cutaneous microvascular NO‐dependent vasodilation is dependent on biological sex and hormonal phase in NHB and NHW young adults. We hypothesized ET_A_R antagonism would increase NO‐dependent vasodilation in NHB females during low hormone phases and males. We further hypothesized that ET_A_R antagonism would increase NO‐dependent vasodilation in NHW females during low hormone phases and males. Finally, we hypothesized the effect of ET_A_R antagonism would have greater effects in NHB participants than NHW participants.

## 2 METHODS

### 2.1 Ethical approval

This study was approved by the Advarra Institutional Review Board (Columbia, MD; No. Pro00024265), the Georgia State University Institutional Review Board, and the United States Food and Drug Administration (IND 138231). All experimental procedures conformed with the Declaration of Helsinki. Each participant provided written and verbal consent before participating in any experimental procedure.

### 2.2 Participants

Participants were recruited from the Georgia State University population and surrounding Atlanta, GA area via (1) flyers posted on and around campus and downtown Atlanta and (2) secondary recruitment. Participant demographics are shown in Table 1. A total of 66 participants were recruited to one of six groups, resulting in 11 individuals per group. Participants included females tested during the low hormone phase of either the natural menstrual cycle or OCP use (OCP; *n* = 22), females tested during the high hormone phase (*n* = 22), and males (*n* = 22). Participants self‐identified as either NHB (*n* = 33) or NHW (*n* = 33).

**TABLE 1:** Participant demographics, hemodynamics, and blood variables.

	Non‐Hispanic Black			Non‐Hispanic White		
	Low hormone phase	High hormone phase	Male	Low hormone phase	High hormone phase	Male
Demographics						
Number, n	11	11	11	11	11	11
Age, years	23 ± 4	22 ± 3	20 ± 2	22 ± 3	23 ± 4	24 ± 6
Height, m	1.7 ± 0.1	1.6 ± 0.1	1.8 ± 0.2	1.6 ± 0.2	1.6 ± 0.1	1.8 ± 0.1
Mass, kg	63 ± 12	65 ± 5	73 ± 13	66 ± 7	58 ± 9	78 ± 10
BMI, kg/m^2^	22 ± 4	24 ± 3	23 ± 3	25 ± 3	23 ± 2	25 ± 4
Hemodynamics						
SBP, mmHg	111 ± 7	110 ± 6	113 ± 6	113 ± 6	110 ± 5	116 ± 6
DBP, mmHg	69 ± 5	68 ± 6	70 ± 5	72 ± 7	69 ± 6	70 ± 8
MAP, mmHg	83 ± 5	82 ± 7	85 ± 8	86 ± 9	83 ± 7	85 ± 10
HR, beats/min	66 ± 8	63 ± 10	62 ± 7	71 ± 8	68 ± 11	61 ± 12
Blood Variables^a^						
HDL, mg/dl	57 ± 16	60 ± 21	51 ± 14	52 ± 14	63 ± 15	43 ± 20
LDL, mg/dl	84 ± 20	91 ± 17	95 ± 17	78 ± 16	83 ± 13	101 ± 26
Total Cholesterol, mg/dl	142 ± 24	150 ± 24	144 ± 19	133 ± 20	142 ± 17	147 ± 21
Glucose, mg/dl	71 ± 28	80 ± 16	67 ± 18	76 ± 21	63 ± 24	86 ± 18
Estradiol, pg/ml^b^	67 ± 58	166 ± 126		49 ± 38	143 ± 118	

*Note*: Values are mean ± SD.

Abbreviations: BMI, body mass index; DBP, diastolic blood pressure; HDL, high density lipoproteins; HR, heart rate; SBP, systolic blood pressure; LDL, low density lipoproteins; MAP, mean arterial pressure.

^a^ Missing data from *n* = 2 non‐Hispanic Black low hormone phase, *n* = 1 non‐Hispanic White low hormone phase, and *n* = 4 non‐Hispanic White males.

^b^ Missing data from *n* = 2 non‐Hispanic Black low hormone phase, *n* = 1 non‐Hispanic White low hormone phase, and *n* = 2 non‐Hispanic White high hormone phase.

The low hormone phase was defined as days 2–5 of the natural menstrual (i.e., menstrual/early follicular phase) cycle or days 22–23 of OCP (i.e., placebo pill phase) (Sims & Heather, 2018). The high hormone phase was defined as days 23–26 of the natural menstrual cycle (i.e., 23–26 days after the onset of menstruation) and days 3+ of OCP (i.e., active pill phase). Although we did not stratify females based on natural menstrual cycle or OCP, we did limit OCP to monophasic OCP and matched the number of females with a natural menstrual cycle and those using OCP in each group, as there is evidence of differences in cutaneous microvascular vasodilation responses between naturally cycling females and females using OCP (Turner, Stanhewicz, Nielsen, Otis, et al., 2023; Turner, Stanhewicz, Nielsen, & Wong, 2023). Use of OCP was limited to any brand/generation of monophasic OCP, but there were no further limitations based on ethinyl estradiol (EE) dose or progesterone generation. Within each group, there were five females (*n* = 5) with a natural menstrual cycle and six females (*n* = 6) using OCP. Phases were determined from self‐report cycle tracking, presentation of OCP pack, and measurement of plasma estradiol (Table 1). Details of the OCPs used by the participants in this study are shown in Table 2. All females were required to submit a urine pregnancy test (McKesson hCG Combo Test Cassette, Consult Diagnostics; Richmond, VA) to confirm negative pregnancy status. A subset of these data (*n* = 10 per group; *n* = 4 NHB females, *n* = 6 NHB males, *n* = 5 NHW females, *n* = 5 NHW males) were published in our previous investigation assessing the effect of ET_A_R inhibition in NHB and NHW young adults (Turner, Hayat, Grosch, Quyyumi, et al., 2023), but was underpowered to assess sex differences. The present study utilized a cross‐sectional design. The effect of NMC/OCP phase was not evaluated by repeated measures due to limitations associated with COVID‐19 (see *Limitations* section below).

**TABLE 2:** Oral contraceptive pill details.

	First generation		Second generation		Fourth generation	
	EE	Progestin	EE	Progestin	EE	Progestin
	0.01 mg	1 mg norethindrone acetate	0.035 mg	0.25 mg norgestimate	0.02 mg	3 mg drospirenone
Non‐Hispanic Black, *n*	1		1		4	
Non‐Hispanic White, *n*	0		1		5	

*Note*: Characteristics of OCPs used by female participants, including generation, ethinyl estradiol (EE) dose, and progestin dose.

All participants were asked to refrain from alcohol, vigorous exercise, and caffeine for at least 8 h before the experimental protocol. All participants were free of cardiovascular, pulmonary, and metabolic diseases and had no history of nerve pain/damage, cancer (chemotherapy or radiation therapy), or skin disorders (e.g., psoriasis) based on self‐report health history. A venous blood sample was analyzed for blood lipids and glucose (Table 1; Alere Cholestech LDX, Abbott Labs, San Diego, CA), and estradiol was measured in blood samples from females (Table 2; Estradiol ELISA; Cayman Chemical, Ann Arbor, MI). Due to technical difficulties with test cassettes and obtaining sufficient blood sample, blood lipid and glucose measurements from seven participants (*n* = 2 NHB females during low hormone phase, *n* = 1 NHW woman during low hormone phase, *n* = 4 NHW males) and estradiol measurements from five participants (*n* = 2 NHB females during low hormone phase, *n* = 1 NHW woman during low hormone phase, *n* = 2 NHW females during high hormone phase) were not obtained.

Eight participants reported having a positive COVID‐19 test, and all reported having minor symptoms (slight cough, headache, cold‐like symptoms). All participants who reported having COVID‐19 participated in this study >6 months after diagnosis, and recent data suggests no effect on cutaneous microvascular function in young adults with minor to moderate COVID‐19 in this timeframe (Dillon et al., 2022).

### 2.3 Instrumentation

Participants were instrumented with two microdialysis fibers (Custom CMA 31 linear probes with a 6 kDa molecular weight membrane; CMA Microdialysis, Harvard Apparatus, Hollister, MA) on the left dorsal forearm as previously described (Miller et al., 2021; Turner et al., 2020; Turner, Hayat, Grosch, Quyyumi, et al., 2023; Turner, Stanhewicz, Nielsen, Otis, et al., 2023; Wong et al., 2020). Microdialysis sites were randomly assigned to receive (1) lactated Ringer's solution (Baxter Healthcare, Deerfield, IL) to serve as a control (Smith et al., 2017) or (2) 500 nM BQ‐123 (Wenner et al., 2011) to serve as an ET_A_R antagonist (AdipoGen Life Sciences, San Diego, CA). All drugs were diluted in sterile lactated Ringer's solution and drawn through filter needles (BD Filter Needle; Becton Dickinson, Franklin Lakes, NJ) or sterile syringe filters (Acrodisc, 13 mm disc, 0.2 μm pore, hydrophilic PES membrane, USP Class VI; Pall Corporation, Port Washington, NY). Trauma from microdialysis fiber placement was allowed to resolve (~45–60 min), and drugs were infused for at least 30 min before the experimental protocol at a rate of 2 μL/min (Beehive Controller and Baby Bee syringe pumps; Bioanalytical Systems, West Lafayette, IN).

To control local skin temperature, the local heater units (VHP1 heater units and VMS‐HEAT controller; Moor Instruments, Axminster, UK) were placed directly over each microdialysis membrane. Integrated laser‐Doppler probes (VP7b probes and VMS‐LDF2 monitor; Moor Instruments) were placed in the center of the local heating unit to obtain red blood cell flux, an index of skin blood flow, at each microdialysis site. Blood pressure was measured at rest and for the duration of the microdialysis experiment from the contralateral (right) arm with the participant in the semirecumbent position using an automated brachial oscillometric device, and heart rate was derived from pulse detection (Welch Allyn Vital Signs Series 6000; Skaneateles Falls, NY). Blood pressure and heart rate measurements were made every 10 min and mean arterial pressure (MAP) was calculated as one‐third pulse pressure plus diastolic pressure. Blood pressure measurements reported in Table 1 are the average of 12–18 measurements (depending on the length of the protocol for each participant).

### 2.4 Experimental protocol

Participants were seated in the semirecumbent position, and the experimental arm was placed at heart level to minimize the effect of hydrostatic pressure on blood flow. Local heater units were first set to 33°C, and baseline skin blood flow was assessed for 8–10 min. Following baseline measurements, local heater temperature was increased from 33°C to 39°C at a rate of 0.1°C/s (Choi et al., 2014). No participants reported pain sensation during the local heating protocol. Once a plateau in skin blood flow was achieved (~30–40 min into local heating), 20 mM L‐NAME (NO synthase inhibitor; Calbiochem, Millipore Sigma, Burlington, MA; catalog #483125) was perfused through both microdialysis sites to quantify NO‐dependent vasodilation (Keen et al., 2015; Miller et al., 2021; Wong et al., 2020; Wong & Fieger, 2010). Once a new plateau following L‐NAME perfusion (i.e., post‐L‐NAME plateau) was achieved at both sites (~30 min into L‐NAME infusion), maximal vasodilation was induced by heating the skin from 39°C to 43°C (0.1°C /s) and infusing 54 mM sodium nitroprusside (Calbiochem, Millipore Sigma, Burlington, MA) (Keen et al., 2015; Miller et al., 2021; Turner et al., 2020; Wong et al., 2020).

### 2.5 Data analysis

Skin blood flow data were continuously recorded at 40 Hz using commercially available hardware and software (PowerLab 16/35 data acquisition and Lab Chart 8 software; ADInstruments, Colorado Springs, CO). Cutaneous vascular conductance (CVC) was calculated as red blood cell flux divided by MAP and standardized to site‐specific maximal vasodilation (%CVC_max_). Baseline data were averaged over a 3‐min period immediately preceding the onset of the local heating protocol. The plateau to local heating was analyzed by averaging a 3‐min period immediately preceding the infusion of L‐NAME, and the post‐L‐NAME plateau was analyzed over a 3‐min period immediately preceding initiation of maximal vasodilation. Maximal skin blood flow was analyzed over a 3‐min period just prior to termination of the protocol. The percent contribution of NO (%NO) to the plateau (Wong & Fieger, 2010) was calculated for both sites.

### 2.6 Statistical analysis

Sample size was determined a priori. Sample size was based on the smallest difference in %NO‐dependent vasodilation between control and BQ‐123 sites, which was observed in NHW females during low hormone phases. Assuming an *α* level of 0.05, 80% power, and %NO‐dependent vasodilation of 63% (SD ± 13%) at control sites and 77% (SD ± 7%) at BQ‐123 sites, the required sample size was 11 per group, or 66 total participants. Skin blood flow (%CVC_max_) and %NO‐dependent vasodilation data were analyzed using a general linear model (i.e., three‐way analysis of variance) with factors for racial identity (NHB and NHW), biological sex (female low hormone, female high hormone, and males), and microdialysis site (control and BQ‐123). Tukey's post hoc test was used to estimate pairwise comparisons. All data were analyzed and graphed using commercially available software (SAS, Cary, NC, and Prism 9, GraphPad Software, Boston, MA). The level of significance was set at *α* = 0.05. Because *p* values do not provide information about the magnitude of observed differences and accepted statistical guidelines are to not report *p* values in isolation (Wasserstein & Lazar, 2016), data are presented as mean ± SD with 95% confidence interval of the difference (95% CI; lower limit, upper limit) and effect sizes (*d*).

## 3 RESULTS

### 3.1 Baseline and maximal data (Table 3)

**TABLE 3:** Baseline and maximal data.

	Non‐Hispanic Black			Non‐Hispanic White		
	Low hormone phase	High hormone phase	Male	Low hormone phase	High hormone phase	Male
Baseline, %CVC_max_						
Control	10 ± 6	13 ± 6	16 ± 8	10 ± 4	16 ± 11	14 ± 7
BQ‐123	11 ± 6	14 ± 5	19 ± 10	10 ± 5	19 ± 14	14 ± 5
Maximal CVC, a.u./mmHg						
Control	2.4 ± 1.1	2.2 ± 1.0	2.6 ± 0.9	2.5 ± 1.0	2.2 ± 0.7	2.3 ± 1.3
BQ‐123	2.2 ± 1.1	2.3 ± 1.5	2.5 ± 1.4	2.4 ± 1.2	2.4 ± 1.6	2.2 ± 1.4

*Note*: Baseline data are expressed as %CVC_max_. For baseline, there was a significant main effect of sex (see the main text for *p* values, 95% CI, and effect sizes). Maximal data is expressed as CVC (laser‐Doppler flux divided by mean arterial pressure; arbitrary units/mmHg). There were no main or interaction effects for maximal CVC. Data are mean ± SD.

For baseline, there was a statistically significant main effect of sex (*p* < 0.01), but the interaction effects were not statistically significant. Baseline was higher in females during the high hormone phase (*p* < 0.01; 95% CI: 1, 9; *d* = 0.76) and males (*p* < 0.01; 95% CI: 2,10; *d* = 0.89) compared with females during the low hormone phase. No difference was found between females during the high hormone phase and males (*p* > 0.99). For maximal CVC, main effects and interaction effects were not statistically significant.

### 3.2 Plateau %CVC_max_

**FIGURE 1:**
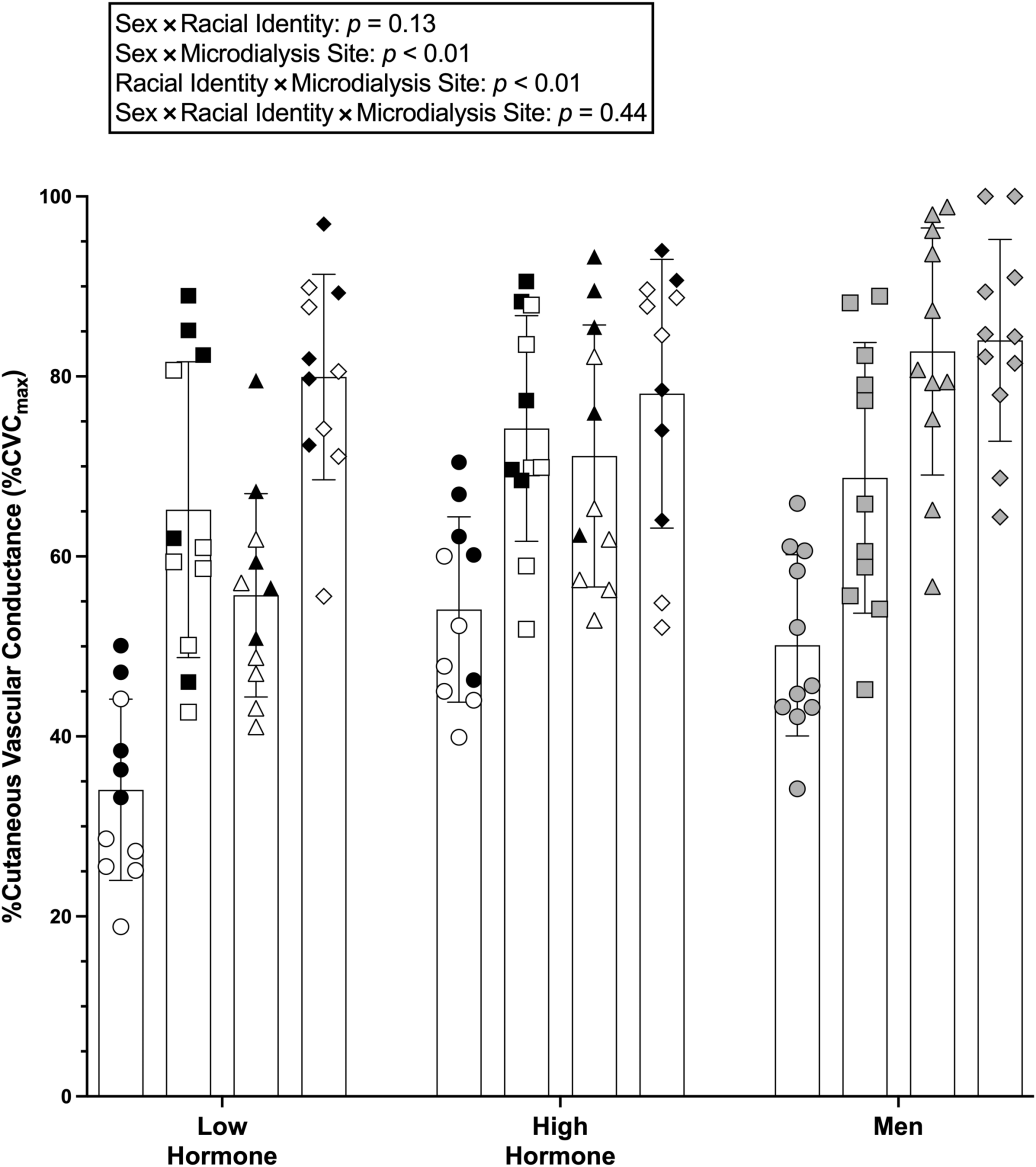
Group plateau (endothelium‐dependent) vasodilation (%CVC_max_) responses shown as mean ± SD (*n* = 11 participants per group, 6 groups; *n* = 66 total). Data for non‐Hispanic Black (NHB) participants are shown as circles for control sites and squares for BQ‐123 sites. Data for non‐Hispanic White (NHW) participants are shown as triangles for control sites and diamonds for BQ‐123 sites. Females with a natural menstrual cycle (NMC) are shown as white symbols, females using oral contraceptive pills (OCP) as black symbols, and males are shown as gray symbols. Data were analyzed with a mixed model with fixed effects of racial identity, biological sex, and microdialysis treatment. Since the three‐way interaction was not statistically significant, symbols for significant comparisons are not shown on the graph. See the main text for exact *p* values, 95% CI, and effect sizes (*d*).

**FIGURE 2:**
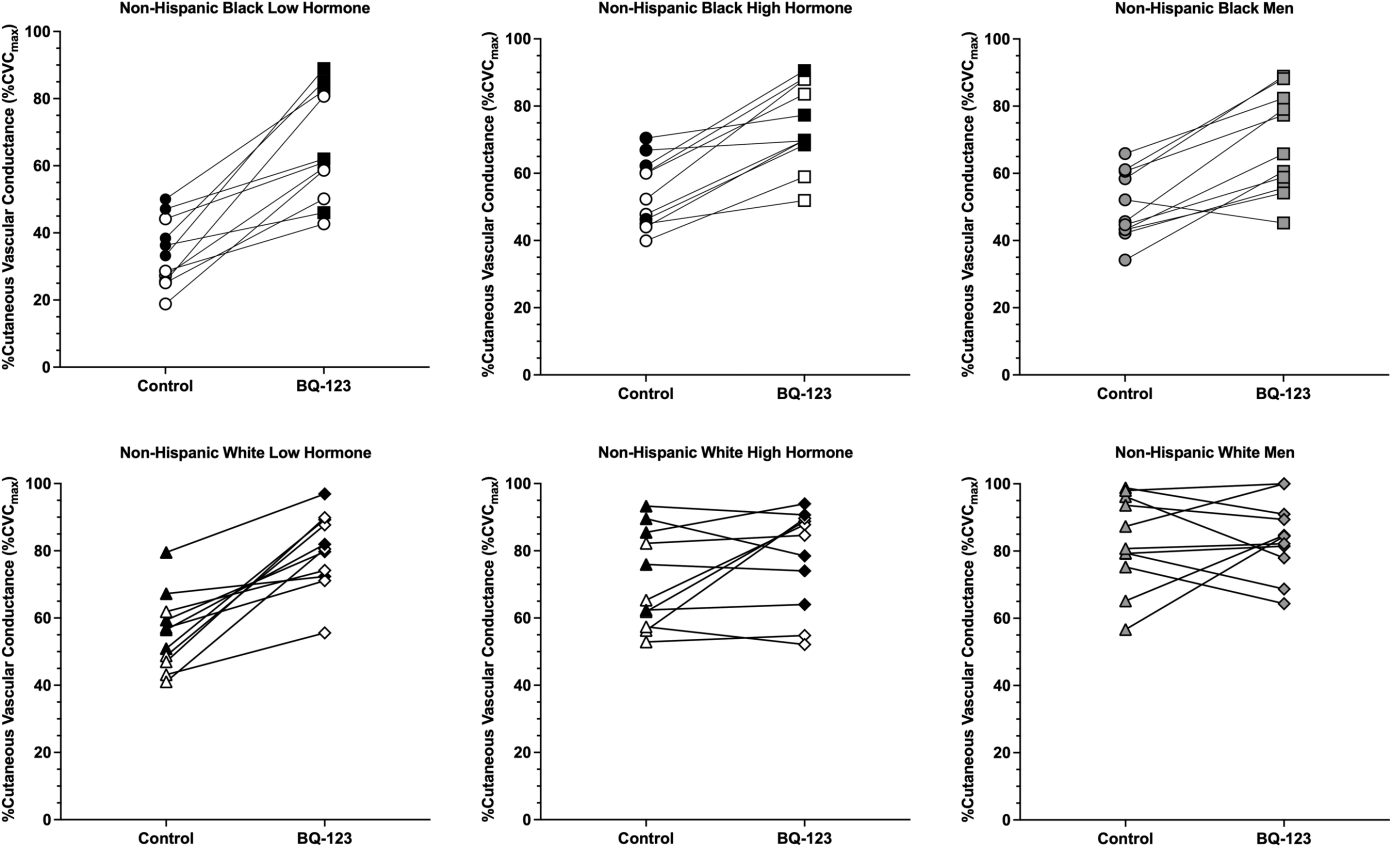
Individual plateau responses to ET_A_R inhibition. Data are shown as the response from control to BQ‐123 sites. Data for non‐Hispanic Black participants (top row) are shown as circles for control sites and squares for BQ‐123 sites. Data for non‐Hispanic White participants (bottom row) are shown as triangles for control sites and diamonds for BQ‐123 sites. Females with a natural menstrual cycle are shown as white symbols, females using OCPs as black symbols, and males are shown as gray symbols. See main text for 95% CI and effect sizes (*d*).

Group mean data for the plateau are shown in Figure 1 and individual responses to ET_A_R inhibition are shown in Figure 2. There was no difference found for the three‐way sex × racial identity × microdialysis site interaction (*p* = 0.44). The following two‐way interactions were statistically significant.

#### 3.2.1 Sex × microdialysis site interactions

At control sites, the plateau was higher in males (66 ± 20 %CVC_max_) compared with females during the low hormone phase (45 ± 15 %CVC_max_) (*p* < 0.01; 95% CI: 12, 31; *d* = 1.2), but no difference was found between males and females during the high hormone phase (63 ± 15 %CVC_max_) (*p* = 0.97; 95% CI: −6, 13; *d* = 0.16). At control sites, the plateau was also higher in females during the high hormone phase compared to the low hormone phase (*p* < 0.001; 95% CI: 8, 27; *d* = 1.2). At BQ‐123 sites, no differences were found between males (76 ± 15 %CVC_max_) and females during the low hormone (73 ± 16 %CVC_max_) (*p* = 0.98; 95% CI: −6, 13; *d* = 0.19) or high hormone (76 ± 14 %CVC_max_) (*p* > 0.99; 95% CI: −9, 10; *d* = 0) phases or between females during the low and high hormone phases (*p* > 0.99; 95% CI: −13, 6; *d* = 0.19). Compared with control sites within group, the plateau was higher at BQ‐123 sites compared to control in males (*p* = 0.01; 95% CI: 2, 18; *d* = 0.6), females during the low hormone phase (*p* < 0.001; 95% CI: 20; 35, *d* = 1.8), and females during the high hormone phase (*p* < 0.001; 95% CI: 6, 21; *d* = 0.9).

#### 3.2.2 Racial identity × microdialysis site interactions

At control sites, the plateau was higher in NHW participants (70 ± 17 %CVC_max_) relative to NHB participants (46 ± 13 %CVC_max_) (*p* < 0.001; 95% CI: 18, 30; *d* = 1.6). The plateau was also higher at BQ‐123 sites in NHW participants (81 ± 13 %CVC_max_) relative to NHB participants (69 ± 15 %CVC_max_) (*p* < 0.001; 95% CI: 5, 18; *d* = 0.9). The plateau was higher at BQ‐123 sites relative to control sites in NHB participants (*p* < 0.00; 95% CI: 17, 30; *d* = 1.6) and NHW participants (*p* < 0.001; 95% CI: 5, 17; *d* = 0.7).

### 3.3 NO‐dependent vasodilation

**FIGURE 3:**
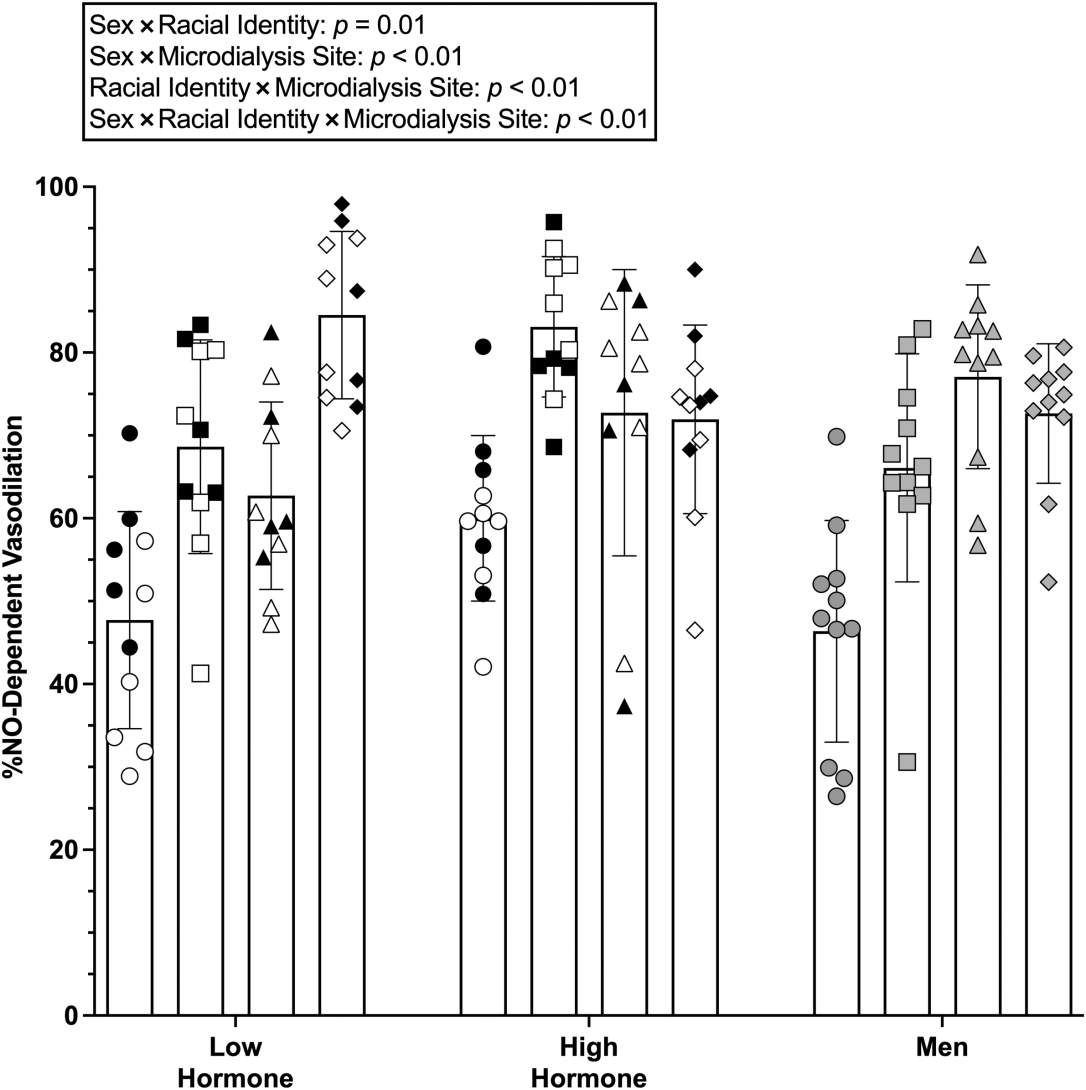
Group NO‐dependent vasodilation shown as mean ± SD (*n* = 11 participants per group, 6 groups; *n* = 66 total). Data for non‐Hispanic Black (NHB) participants are shown as circles for control sites and squares for BQ‐123 sites. Data for non‐Hispanic White (NHW) participants are shown as triangles for control sites and diamonds for BQ‐123 sites. Females with a natural menstrual cycle (NMC) are shown as white symbols, females using oral contraceptive pills (OCP) as black symbols, and males are shown as gray symbols. Data were analyzed with a mixed model with fixed effects of racial identity, biological sex, and microdialysis treatment. There was a significant three‐way interaction. a, *p* ≤ 0.05 versus respective control site; b, *p* ≤ 0.05 non‐Hispanic Black females during high hormone phase versus non‐Hispanic Black males at BQ‐123 sites; c, *p* ≤ 0.05 non‐Hispanic Black males versus non‐Hispanic White males at control sites. See the main text for exact *p* values, 95% CI, and effect sizes (*d*).

**FIGURE 4:**
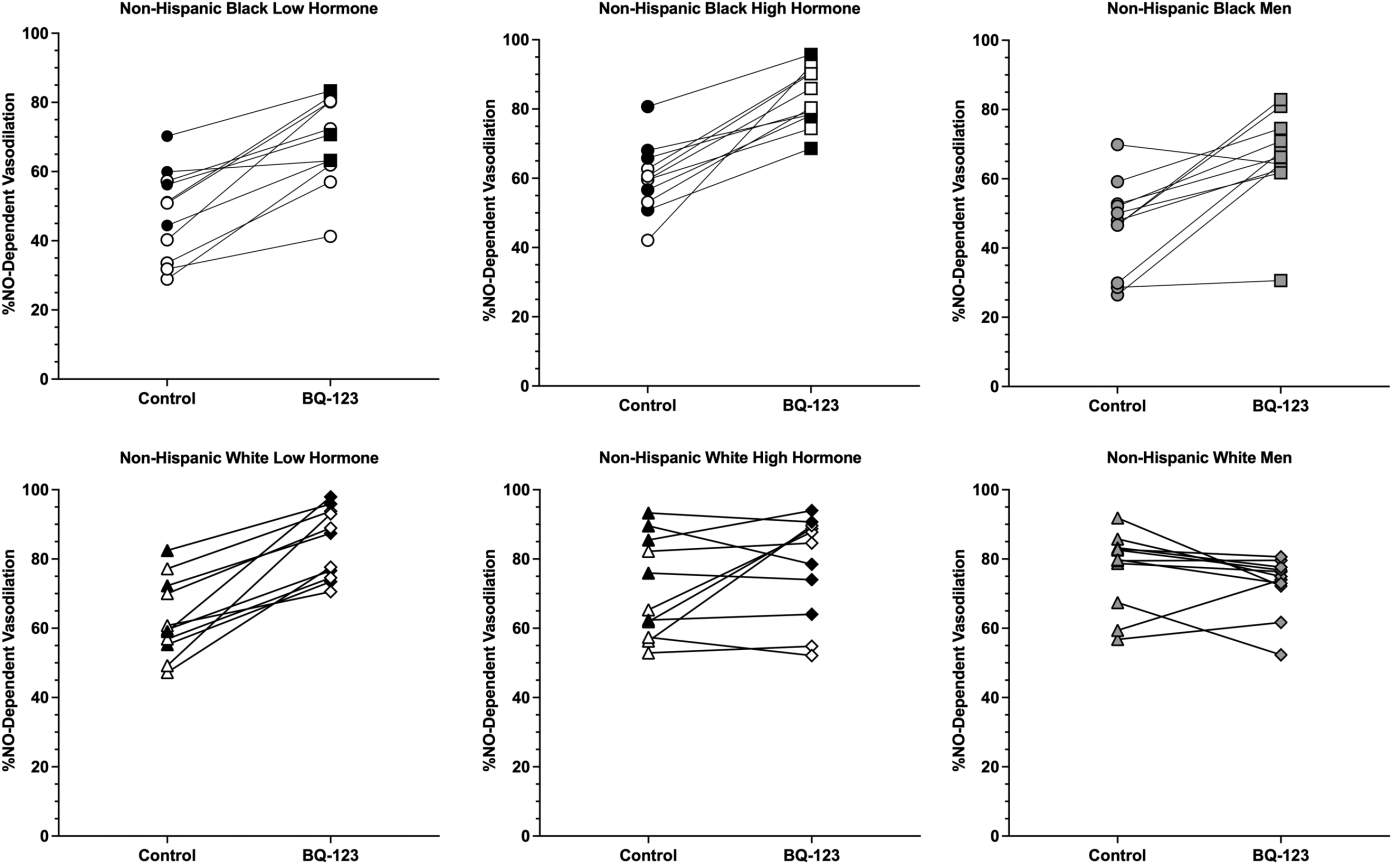
Individual NO‐dependent vasodilation responses to ET_A_R inhibition. Data are shown as the response from control to BQ‐123 sites. Data for non‐Hispanic Black participants (top row) are shown as circles for control sites and squares for BQ‐123 sites. Data for non‐Hispanic White participants (bottom row) are shown as triangles for control sites and diamonds for BQ‐123 sites. Females with a natural menstrual cycle are shown as white symbols, females using OCPs as black symbols, and males are shown as gray symbols. See the main text for 95% CI and effect sizes (*d*).

Group mean data for NO‐dependent vasodilation are shown in Figure 3 and individual responses to ET_A_R inhibition are shown in Figure 4. There was a statistically significant three‐way sex × racial identity × microdialysis site interaction (*p* < 0.01).

#### 3.3.1 Non‐Hispanic Black participants

At control sites, NO‐dependent vasodilation was 48 ± 13 %NO in females during the low hormone phase, 60 ± 10 %NO in females during the high hormone phase, and 46 ± 13 %NO in males. At control sites, there were no differences found between females during low and high hormone phases (*p* = 0.41; 95%CI: −29, 45; *d* = 1.03), females during low hormone phases and males (*p* > 0.99; 95% CI: −16, 18; *d* = 0.15) or females during high hormone phases and males (*p* = 0.26; 95% CI: −3, 31; *d* = 1.21).

At BQ‐123 sites, NO‐dependent vasodilation was 69 ± 13 %NO in females during the low hormone phase, 82 ± 8 %NO in females during the high hormone phase, and 66 ± 14 %NO in males. There was no difference found between females during low and high hormone phases (*p* = 0.18; 95% CI: −32, 3; *d* = 1.20). NO‐dependent vasodilation was greater in females during the high hormone phase compared to males (*p* = 0.05; 95%: CI: 0, 34; *d* = 1.40). There was no difference found between females during the low hormone phase and males (*p* > 0.99; 95% CI: −15, 20; *d* = 0.22). Compared to control sites, BQ‐123 increased NO‐dependent dilation in females during the low (*p* < 0.01; 95% CI: −34, −7; *d* = 1.62) and high hormone phases (*p* < 0.01; 95% CI: −37, −10; *d* = 2.40), and in males (*p* < 0.01; 95% CI: −33, −6; *d* = 1.48).

#### 3.3.2 Non‐Hispanic White participants

At control sites, NO‐dependent dilation was 63 ± 11 %NO in females during the low hormone phase, 73 ± 17 %NO in females during the high hormone phase, and 77 ± 11 in males. There was no difference found between females during high and low hormone phases (*p* = 0.72; 95% CI: −27, 7; *d* = 0.70), between females during the low hormone phase and males (*p* = 0.19; 95% CI: −31, 3; *d* = 1.27), or between females during the high hormone phase and males (*p* > 0.99; 95% CI: −21, 13; *d* = 0.28).

At BQ‐123 sites, NO‐dependent dilation was 85 ± 10 %NO in females during the low hormone phase, 72 ± 12 %NO in females during the high hormone phase, and 73 ± 8 %NO in males. There were no differences found between females during low and high hormone phases (*p* = 0.38; 95% CI: −5, 30; *d* = 1.18), females during the low hormone phase and males (*p* = 0.47; 95% CI: −5, 29; *d* = 1.33), or females during the high hormone phase and males (*p* > 0.99; 95% CI: −18, 16; *d* = 0.09). Compared to control sites, BQ‐123 increased NO‐dependent dilation in females during the low hormone phase (*p* < 0.01; 95% CI: −35, −8; *d* = 2.09). There was no difference found between control and BQ‐123 sites in females during the high hormone phase (*p* > 0.99; 95% CI: −13, 14; *d* = 0.07) or males (*p* > 0.99; 95% CI: −9, 18; *d* = 0.42).

#### 3.3.3 Comparisons between non‐Hispanic Black and White participants

During the low hormone phase, there was no difference found in NO‐dependent dilation between NHB females and NHW females at either control (*p* = 0.14; 95% CI: −32, 2; *d* = 1.25) or BQ‐123 (*p* = 0.09; 95% CI: −33, 1; *d* = 1.38) sites, but there were large effect sizes. During the high hormone phase, there were no differences found between NHB and NHW females at either control (*p* = 0.36; 95% CI: −30, 4; *d* = 0.93) or BQ‐123 (*p* = 0.57; 95% CI: −6, 28; *d* = 0.98) sites, but there were large effect sizes. In males, NO‐dependent dilation was lower in NHB compared to NHW males at control (*p* < 0.01 95% CI: −48, 14; *d* = 2.57) but not BQ‐123 (*p* = 0.98; 95% CI: −24, 10; *d* = 0.61) sites.

## 4 DISCUSSION

The overall finding of this study is that ET_A_R inhibition can increase microvascular function in both NHB and NHW young adults, but this effect depends, in part, on biological sex, and the menstrual cycle/OCP phase. Consistent with previous data (Akins et al., 2022), we found that inhibition of ET_A_R increases endothelium‐dependent vasodilation (i.e., local heating plateau; Figures 1 and 2) and NO‐dependent vasodilation (Figures 3 and 4) in young NHB females in the low hormone phase of the natural menstrual cycle or OCP use. The present data extend these findings to show a similar effect during the high hormone phase and in young NHB males. The present data also suggest ET_A_R inhibition can increase NO‐dependent vasodilation in young NHW females during low hormone phases of either the natural menstrual cycle or OCP use. Conversely, ET_A_R inhibition had no effect on NO‐dependent vasodilation in young NHW males or NHW females during the high hormone phase of the natural menstrual cycle or OCP use.

### 4.1 Plateau %CVC_max_ data

The plateau to local heating is representative of general microvascular endothelial function. Results from the present study are consistent with previous data showing a decreased plateau at control sites in all three groups of young, healthy NHB adults relative to NHW young adults (Hurr et al., 2018; Kim et al., 2018; Miller et al., 2021; Patik et al., 2018; Turner et al., 2020; Turner, Hayat, Grosch, Quyyumi, et al., 2023; Wong et al., 2020). In this study, we also show that microvascular endothelial function is lowest in NHB and NHW females during low hormone phases relative to males and females during high hormone phases in both groups. This finding is similar to recent work published by our lab (Turner, Stanhewicz, Nielsen, Otis, et al., 2023) but extends these findings to include a comparator group of females in high hormone phases. These data also suggest that ET_A_R activity may be enhanced in females when female sex hormone levels are low, as ET_A_R inhibition with BQ‐123 increased endothelial function in females in low hormone phases, such that there was no difference in plateau compared with females in high hormone phases or males with the treatment. Whether other pathways interact with ET_A_R and modulate the plateau across menstrual cycle/contraceptive pill phase is unknown. The EDHF pathway is known to contribute to the local heating response (Brunt & Minson, 2012) and it is possible that alterations in the EDHF pathway coincide with changes in ET_A_R activity across the menstrual cycle/contraceptive pill phase, but this has yet to be investigated.

At BQ‐123 sites, microvascular function was increased compared to control sites in both NHB and NHW participants, indicating there is ET_A_R activity in both groups of young, otherwise healthy individuals; however, the response to BQ‐123 was lesser in NHW participants compared with NHB participants overall. There was also an increase in microvascular function at BQ‐123 sites, relative to control, in all three biological sex groups. However, because there was not a significant three‐way interaction (sex × racial identity × microdialysis site), it is uncertain whether there were different responses to ET_A_R inhibition between NHB and NHW females and males.

### 4.2 NO‐dependent vasodilation data

The effect of ET_A_R inhibition on NO‐dependent vasodilation appears to be independent of biological sex and natural menstrual cycle/OCP phase in NHB young adults, while it is dependent on biological sex and natural menstrual cycle/OCP phase in NHW young adults. There was no effect of ET_A_R inhibition in NHW males or females during high hormone phases, but there was a robust increase in NO‐dependent vasodilation in NHW females during the low hormone phases. Thus, in NHW females, low levels of female sex hormones (presumably estradiol) enhance ET_A_R activity in the microvasculature. These data agree with other studies that have shown estradiol regulates ET_A_R and ET_B_R responses in the microvasculature in young females (Sebzda et al., 2018; Shoemaker et al., 2021) and that ET_A_R inhibition augments both endothelium‐dependent and NO‐dependent vasodilation in NHB and NHW young females during low hormone phases (Akins et al., 2022). Previous work suggests that neither ET_B_R inhibition nor L‐arginine infusion affects NO‐dependent vasodilation in young Black or White females during low hormone phases (Akins et al., 2022), suggesting that neither constrictive ET_B_R on VSMC, vasodilator ET_B_R on endothelial cells, nor reduced NO substrate contribute to the reduction in NO‐dependent vasodilation during low hormone phases.

Given that ET_A_R inhibition had no effect on NO‐dependent vasodilation in NHW females during the high hormone phase suggests high levels of female sex hormones suppress the effects of ET_A_R in young NHW females. Conversely, there appears to be residual effects of ET_A_R in NHB females during the high hormone phase, as evidenced by the additional increase in NO‐dependent vasodilation with BQ‐123. However, there was no observed difference in estradiol concentrations in NHB and NHW female participants in this study. Interestingly, during the high hormone phase, ET_A_R inhibition increased NO‐dependent vasodilation in NHB females to a level that was greater than that observed in NHW females, suggesting a potential additive effect of ET_A_R inhibition and female sex hormones in NHB females. It is possible that ET_A_R inhibition unmasks a role for ET_B_R‐mediated vasodilation in NHB females during the high hormone phase. This would be reflective of similar previous findings (Sebzda et al., 2018; Shoemaker et al., 2021). However, as ET_A_R inhibition augmented NO‐dependent vasodilation in NHB females during low and high hormone phases, it is also possible that estradiol is not a primary mediating factor of the relationship between ET_A_R and NO‐dependent vasodilation in NHB females. Other factors may have a greater influence on this relationship in NHB females; potential targets may include, but are not limited to, angiotensin II (Ferri et al., 1999; Lin et al., 2014), cortisol (Kanse et al., 1991; Lopez et al., 2020), or insulin (Ferri et al., 1995; Oliver et al., 1991), as they are evidenced to interact with the ET‐1 system in the vasculature and clinical evidence supports may be of importance (Fuller‐Rowell et al., 2019; Jeong et al., 2019).

The role of ET_A_R in NO‐dependent vasodilation is, overall, independent of biological sex and female hormonal phase in NHB young adults. This suggests that ET_A_R activation is upregulated in NHB compared with NHW young adults overall, and that this is a conserved mechanism of microvascular dysfunction between sexes. Previous studies have highlighted sex‐specific mechanisms that contribute to reduced microvascular function in NHB young adults, such that NADPH and xanthine oxidase contribute to reduced function in young Black males but not females (Patik et al., 2018). It is possible that these additional male‐specific mechanisms in NHB young adults contribute to the presentation of greater constrictor action toward ET‐1 relative to females. Therefore, investigating other mediators or additive effectors of ET‐1 synthesis and receptor function is further warranted in NHB males and females.

### 4.3 Baseline %CVC_max_ data

Baseline %CVCmax was observed to be lower in females during low hormone phases compared with females during high hormone phases and males. There are limited studies assessing cutaneous microvascular function across low and high hormone states in females. However, in a recent publication from our lab (Turner, Stanhewicz, Nielsen, Otis, et al., 2023), we also observed lower baseline blood flow in females in low hormone phases (naturally menstruating and OCP placebo phase grouped together and analyzed separately) compared with males. A prior publication suggests greater sympathetic regulation of basal cutaneous blood flow in females compared with males (Cooke et al., 1990). Based on these previous findings and the current findings, it is possible females in low hormone phases may experience an upregulation of sympathetic control compared with males, but that during phases with higher estradiol, this is withdrawn. Alternatively, this could also be related to changes in basal body temperature across the hormonal phase, but this has yet to be directly assessed.

### 4.4 Implications

Several lines of evidence implicate ET‐1 and the ET_A_R subtype in the pathogenesis of vascular dysfunction in NHB individuals. Compared with NHW adults, NHB adults have greater resting plasma ET‐1 levels, ET‐1 generation in response to acute psychological and physiological stressors, ET‐converting enzyme protein levels, quantity of ET_A_R, and ratio of ET receptors located on VSMC versus on endothelial cells (leading to greater constrictor versus dilator action of ET‐1) (Grubbs et al., 2002; Treiber et al., 2000). Hypertensive NHB adults also exhibit greater ET_A_R subtype‐dependent vasoconstriction than hypertensive NHW counterparts (Campia et al., 2004). As such, the present data further support a specific mechanism for ET_A_R in blunted vasodilator responses in both NHB males and females.

There has also been debate in the literature as to whether it is appropriate to control for menstrual cycle and/or OCP phase (Stanhewicz & Wong, 2020; Wenner & Stachenfeld, 2020). The data from the present study suggests either controlling for menstrual cycle/OCP phase or testing at multiple time points is important and may reveal mechanistic insights that may not otherwise be observed. However, we recognize that this is not always feasible and should be determined, a priori, by the research question.

### 4.5 Limitations

The following limitations warrant consideration. First, social determinants of health (SDoH) are important for not just cardiovascular health but overall health. In this study, we did not assess SDoH. Clinically meaningful assessments of SDoH generally require much larger sample sizes. For example, a recent analysis of a racially and ethnically diverse sample of 3590 adolescents from NHANES found variable associations between SDoH and cardiovascular health, ranging from strong to no associations (Connolly et al., 2022). Since SDoH can influence physiological responses, a large, prospective study powered to assess associations between SDoH and NO‐dependent dilation is warranted. Second, we did not restrict the inclusion of females to either naturally cycling or OCP use. It is possible endogenous and exogenous female sex hormones interact with the endothelin system differently, and the results could be influenced by having heterogenous groups of females. Third, we did not restrict OCP use to a single formulation and different formulations/generations of OCP may have different effects on the endothelin system. Differing doses of EE (low vs. very low dose) and different generations of progesterone may have different effects on vascular function, which could be due to their effects on the endothelin system. Fourth, due to technical difficulties, we do not have complete blood profiling data on all participants. It is possible some of the participants had elevated lipids and/or glucose, but no participants reported having a diagnosis of type 1 or 2 diabetes, hypercholesterolemia, or dyslipidemia, and the magnitude of responses in both groups was similar to results of previous studies where glucose and lipids were analyzed (Hurr et al., 2018; Patik et al., 2018). It is also possible levels of female sex hormones in females with missing blood samples were not within typical limits for either low or high hormone phases, though neither statistical analysis of the data nor interpretation of the data was different when data from these participants were removed; therefore, data from these participants was included. All naturally cycling females in this study tracked their cycle and confirmed having consistent cycles for at least the past 2 months, and all females using OCP confirmed regular usage with no missed days. Fifth, we did not investigate whether microvascular responses to ET_B_R differ by racial identity and/or biological sex. The activity of ET_B_R does appear to be increased in females during high hormone phases (Sebzda et al., 2018; Shoemaker et al., 2021), but it is unclear whether ET_B_R activity differs between NHB and NHW individuals. Since there are currently no data investigating the ET‐1 system in NHB and NHW females during both low and high hormone phases, we chose to focus on the ET_A_R since this specific receptor has been shown to contribute to reduced microvascular function in NHB individuals. Finally, a repeated measures design for female participants would have been a stronger experimental design in this study. A repeated measures design was originally planned for this experiment, and data collection for this study began prior to the COVID‐19 pandemic and subsequent lab closure. Thus, follow‐up for most females who participated prior to COVID‐19 was not possible. Rather than discarding data collected prior to COVID‐19, the experimental design for this study was changed to a cross‐sectional approach to best utilize both material resources and participant effort.

## 5 CONCLUSIONS

Data from the present study contribute to the line of evidence supporting a role for ET‐1 and the ET_A_R subtype in the reduction of endothelium‐dependent and NO‐dependent vasodilator function in NHB adults. Importantly, this effect was significant in NHB females (in low and high hormonal phases) and males, indicating a consistent influence of the pathway on microvascular function in NHB adults. This study also demonstrates an effect of ETAR activity on endothelium‐dependent and NO‐dependent vasodilation in NHW females during a low but not high hormone phase, which contributes to the understanding of sex hormones on ET‐1 and ET_A_R function. Overall, ET_A_R may be an underlying mechanism contributing to disparities in cardiovascular health outcomes between NHB and NHW individuals. Overall, these findings support a role for ET_A_R in endothelium‐dependent and NO‐dependent vasodilation, which is influenced by both biological sex and racial identity.

## FUNDING INFORMATION

This study was funded by NIH grant R01 HL141205 (BJW).

## CONFLICT OF INTEREST STATEMENT

The authors have no conflict of interest to report.

## Data Availability

Data are available upon reasonable request.
